# [Corrigendum] Tanshinol upregulates the expression of aquaporin 5 in lung tissue of rats with sepsis

**DOI:** 10.3892/ol.2026.15649

**Published:** 2026-05-14

**Authors:** Jianfeng Xu, Lei Yang, Liang Dong

Oncol Lett 16: 3290–3296, 2018; DOI: 10.3892/ol.2018.9026

Following the publication of the above paper, it was drawn to the Editor's attention by a concerned reader that, regarding the hematoxylin and eosin-stained images shown in Fig. 3 on p. 3293, the data panels for Fig. 3A (the Ctrl group) and Fig. 3B (the SO group) contained an overlapping section, suggesting that these data panels, which were intended to show the results of differently performed experiments, had been derived from the same original source.

After re-examining their original data, the authors have realized that the data in Fig. 3A for the control group were inadvertently included in this figure incorrectly. The revised version of Fig. 3, now showing the correct data for the control group in Fig. 3A, is shown below. Note that this error did not affect any other data, the results or the conclusions reported in the study. The authors are grateful to the Editor of *Oncology Letters* for allowing them this opportunity to publish a Corrigendum, and all the authors agree with its publication. Furthermore, the authors apologize to the readership for any inconvenience caused.

## Figures and Tables

**Figure 3.**
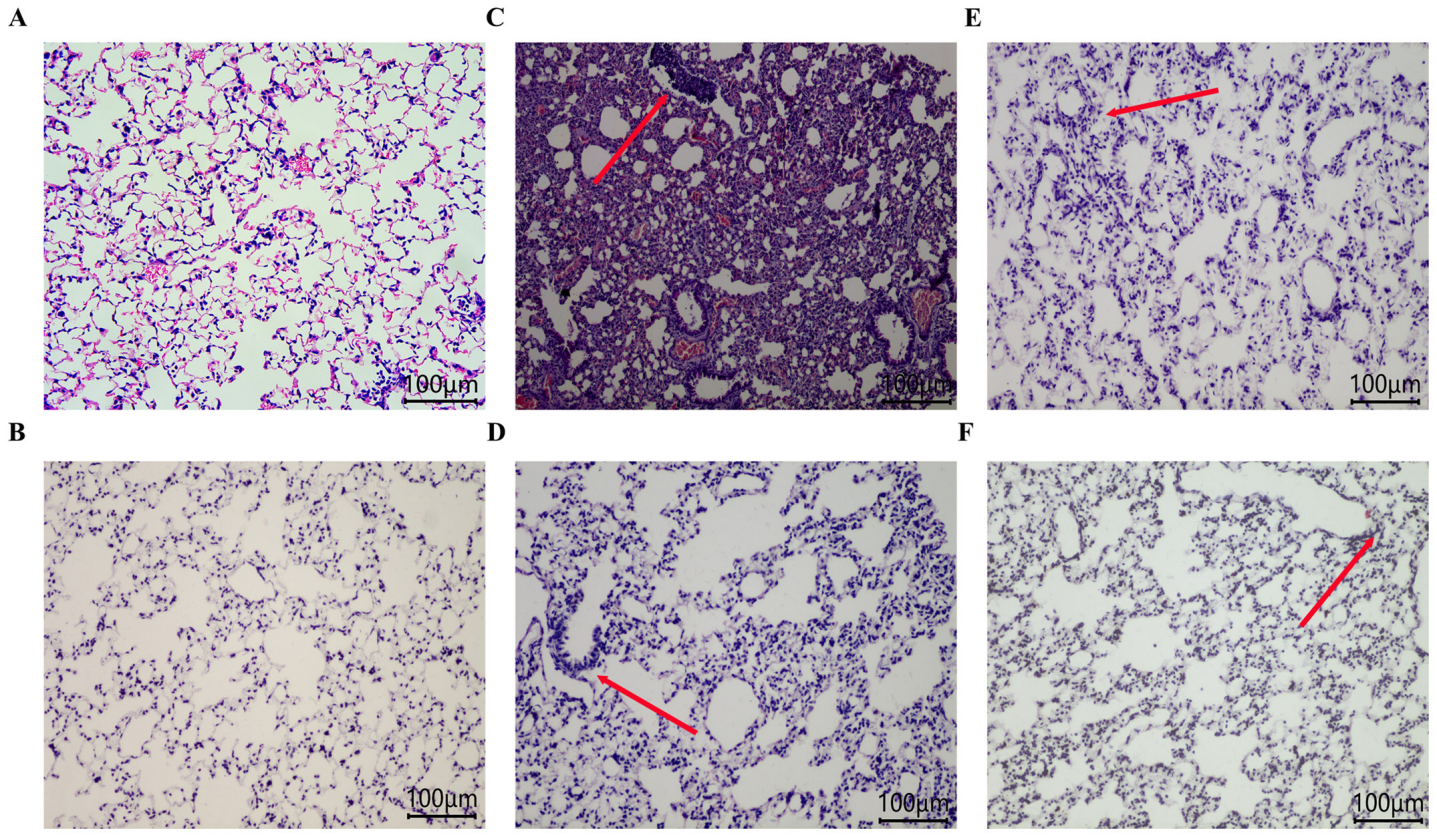
Pathological imaging of rat lung tissue (magnification, ×100) following hematoxylin and eosin staining. (A) ctrl group; (B) SO group; (C) Sepsis group; (D) Tans-L group; (E) Tans-M group; (F) Tans-H group. Red arrows indicate inflammatory cell infiltration. ctrl, control; SO, sham operation group; Sepsis, model group; Tan-L, low dose tanshinol group (5 mg/kg); Tan-M, moderate dose tanshinol group (10 mg/kg); Tan-H, high dose tanshinol group (20 mg/kg).

